# A Neuropeptide Y Variant (rs16139) Associated with Major Depressive Disorder in Replicate Samples from Chinese Han Population

**DOI:** 10.1371/journal.pone.0057042

**Published:** 2013-02-27

**Authors:** Yongjun Wang, Yutao Yang, Li Hui, Changle Tie, Feng Li, Zhi-Qing David Xu, Chuanyue Wang

**Affiliations:** 1 Beijing Anding Hospital, Capital Medical University, Beijing, China; 2 Beijing Key Laboratory of Brain Major Disorders, Capital Medical University, Beijing, People's Republic of China; 3 Tianjin Anding Hospital, Tianjin, China; Baylor College of Medicine, United States of America

## Abstract

**Objective:**

This study aimed to investigate the single nucleotide polymorphisms (SNPs) of neuropeptide Y (NPY) and major depressive disorder (MDD) in Chinese Han population.

**Design:**

Prospective and randomized studies were carried out.

**Patients:**

A total of 700 patients (324 male and 376 female; mean age = 40±14.9 years) with depression who met the diagnostic criteria of Diagnostic and Statistical Manual of Mental Disorders, Fourth Edition (DSM-IV) and 673 healthy controls (313 male and 360 female; mean age = 41.9±17.2 years) were used to investigate the relationship between SNPs of NPY and the pathogenesis of MDD. A total of 417 patients (195 male and 202 female; mean age = 36±14.2 years) diagnosed with MDD and 314 healthy controls (153 male and 161 female; mean age = 37.9±14.2 years) from Chinese Han population were used to verify the relationship between SNPs of NPY and the pathogenesis of MDD.

**Intervention and outcome:**

Ligase detection reactions were performed to detect the SNP sites of NPY. A series of statistical methods was carried out to investigate the correlation between the NPY gene SNP and MDD.

**Results:**

Statistical analysis showed a significant correlation between the SNP sites rs16139 in NPY and the morbidity of depression. Patients with MDD have a lower frequency of A-allele in rs16139 in replicate samples from Chinese Han population. However, the frequency varied between male and female patients.

**Conclusion:**

The gene polymorphism loci rs16139 was closely related to MDD in Chinese Han population.

## Introduction

Major depressive disorder (MDD) is a mental disorder characterized by an extremely low mood accompanied by low self-esteem, and by loss of interest or pleasure in normally enjoyable activities. About 10% to 15% of the general population is estimated to experience clinical depression during their lifetime [1]. In the United States, around 3.4% of persons with major depression commit suicide, and up to 60% of persons who commit suicide have depression or another mood disorder [2]. Family, twin, and adoption studies indicate that genetic factors play important roles in the development of MDD [3]. Twin studies suggest a heritability of 40% to 50% and family studies indicate a twofold to threefold increase in the lifetime risk of developing MDD among first-degree relatives [4]. Interventional and preventive measures play important roles in preventing the onset of depression. Therefore, effective early diagnosis strategies, such as genetic testing, are particularly important. However, the diagnosis of MDD is complicated. It is based on a patient's self-reported experiences, behaviors reported by relatives or friends, and a mental status examination. Several studies have shown that the expression level of neuropeptide Y (NPY) gene in the brain is closely related to the onset of depression. Thus, NPY may be a promising target gene for the early diagnosis of MDD.

Neuropeptides are expressed and released by neurons. They mediate or modulate neuronal communication by acting on cell surface receptors. Numerous studies have shown that the brain molecule NPY helps restore calmness after stressful events. In Michigan, several scientists have found that persons with low levels of NPY in their brain may be at higher risk of suffering from depression [5]. Previous studies in mice have reported that high levels of NPY exert an anti-stress effect and reduce emotional responses [6]. Moreover, low levels of NPY in humans are associated with poor responses to antidepressant therapy [7].

In this work, single gene polymorphisms (SNPs) of NPY in patients with MDD and healthy controls were investigated to evaluate the genetic risk factors for MDD.

## Materials and Methods

### Participants

A total of 700 patients (324 male and 376 female; mean age = 40±14.9 years) diagnosed with MDD according to the Hamilton anxiety scale and diagnostic criteria, as well as 673 healthy controls (313 male and 360 female; mean age = 41.9±17.2 years) from Chinese Han population were used to investigate the relationship between SNPs of NPY and pathogenesis of MDD. About 417 patients (195 male and 202 female; mean age = 36±14.2 years) diagnosed with MDD and 314 healthy controls (153 male and 161 female; mean age = 37.9±14.2 years) from Chinese Han population were used to verify the relationship between SNPs of NPY and the pathogenesis of MDD. All cases completed the same diagnostic instrument, i.e., the Structured Clinical Interview for DSM IV-I (SCID-I), and met the MDD criteria of Diagnostic and Statistical Manual of Mental Disorders, Fourth Edition (DSM-IV). Healthy individuals without history of psychosis screened by SCID-I were recruited from among university and hospital employee, as well as community inhabitants in Beijing and XinXiang cities. All subjects signed an informed consent form. This study was approved by the Ethics Committee of the Beijing AnDing Hospital and XinXiang Medical University.

### Methods

DNA isolation and amplification. Genomic DNA was extracted from whole blood using a genomic DNA Purification Kit (TAKARA). Genotyping for polymorphisms was conducted by polymerase chain reaction (PCR) followed by ligase detection reaction (LDR). A series of PCRs was performed to amplify the gene fragments of NPY, which contained each of the SNP sites listed in supplemental table S1 and supplemental table S2. All PCR primers and gene probes used in PCR and LDR were designed using Primer 5.0 and Oligo 6.0 software and synthesized by Shanghai Biowing Applied Biotechnology Company. The PCRs were performed at 94°C for 2 min; followed by 35 cycles of 30 s at 94°C, 20 s at 59°C, 62°C or 53°C, and 60 s at 72°C; and a final extension at 72°C for 3 min.

LDR. LDRs were performed in 20 μL of buffer containing 20 mM Tris–HCl (pH 7.6), 10 mM MgCl_2_, 100 mM KCl, 10 mM DTT, 1 mM EDTA, 1 mM NAD^+^, 12.5 nM of each probe, 1 μL of PCR product, and 0.1 mM DNA ligase. LDR was performed on a MJ PCT-200 gradient thermal cycler by incubating at 95°C for 2 min and then cycling for 35 cycles at 94°C for 30 s and 60°C for 2 min. The reaction was stopped by adding 0.5 mL of 0.5 mM EDTA. About 1 μL of the LDR products was mixed with an equal volume of ABI GS-500 ROX (fluorescent labeling molecular weight standard) and deionization formamide. The mixture was denatured at 95°C for 2 min, rapidly chilled on ice before loading on a 5% polyacrylamide and 5 M urea gel, and then electrophoresed for 2.5 h at 3000 V (377 DNA Prism Sequencer; Applied Biosystems). Genemapper software was used to analyze the ligation products.

### Data analysis

All analyses were performed using SPSS software program version 16.0 for Windows (SPSS Institute Inc.). The chi-squared test was used to evaluate differences in genotypic frequency distributions for the genotype of NPY polymorphisms between the MDD cases and controls. The Hardy–Weinberg equilibrium (HWE) test was also performed to explore the departure. The genotype data for each polymorphism were further divided based on sex. The associations between the combined genotypes and haplotypes of NPY polymorphisms were evaluated by multivariate logistic regression analysis. All statistical tests were two-sided, and *p* < 0.05 was considered statistically significant.

## Results

### SNP detection and HWE test

We selected 10 NPY SNPs in this study, and the success rate of genotyping was >97.1%. Only the C-allele was detected in rs695110 and the genotype was CC; no other genotype was detected. As shown in supplemental table S3, all alleles and genotypes of the selected 10 NPY SNPs were in HWE except for rs16478 (*p*>0.05).

### Allele and genotypic frequency distribution

Table 1 shows the analysis of the relationship between NPY SNPs and the susceptibility of MDD. The results indicated that the genotype of rs16139 may be correlated with MDD. The A/A genotype had a lower frequency in rs16139 in the patient group with MDD, but the A/G genotype displayed a significantly increased frequency (*χ* ^2^ = 22.683, *p* = 0.00000196). Compared with the healthy controls, patients with MDD showed a lower frequency of A-allele and a higher G-allele in rs16139 (OR = 0.039, 95% CI = 0.005−0.289, *χ* ^2^ = 22.477, *p* = 0.000002). After adjusting with the Bonferroni correction and permutation test (10 000 times), the results were still significant (*p* = 0.0018 and 0.01). The Bonferroni correction was also used to adjust for the number of statistical tests.

**Table 1:** The correlation analysis between the Genotype and Alleles of NPY SNPs and the susceptibility of MDD.

SNPs ID	group	Genotype			P	Allele		OR	95%CI	P
rs16147	MDD	C/C(0.467)	C/T(0.431)	T/T(0.102)	0.723	C(0.682)	T(0.318)	0.943	0.80∼1.111	0.483
	Con	0.479	0.431	0.090		0.695	0.305			
rs16478	MDD	C/C(0.555)	C/T(0.385)	T/T(0.061)	0.931	C(0.747)	T(0.253)	1.004	0.843∼1.197	0.957
	Con	0.550	0.393	0.057		0.746	0.254			
rs16139	MDD	A/A(0.961)	A/G(0.039)		1.96E−006	A(0.981)	G(0.019)	0.039	0.005∼0.289	2.22E−006
	Con	0.998	0.002			0.999	0.001			
rs16138	MDD	C/C(0.061)	C/G(0.366)	G/G(0.573)	0.903	C(0.224)	G(0.756)	1.041	0.870∼1.245	0.659
	Con	0.056	0.361	0.583		0.236	0.764			
rs3025118	MDD	G/G(0.946)	G/T(0.054)		0.200	G(0.973)	T(0.027)	0.722	0.435∼1.198	0.206
	Con	0.960	0.040			0.980	0.020			
rs16135	MDD	C/C(0.507)	C/T(0.407)	T/T(0.085)	0.757	T(0.289)	C(0.711)	1.065	0.902∼1.259	0.456
	Con	0.490	0.416	0.094		0.302	0.698			
rs5574	MDD	C/C(0.383)	C/T(0.474)	T/T(0.143)	0.973	C(0.620)	T(0.308)	1.013	0.866∼1.186	0.868
	Con	0.377	0.480	0.143		0.617	0.383			
rs6951110	MDD	C/C(1.000)				C(1.000)				
	Con	1.000				1.000				
rs16129	MDD	G/G(0.468)	G/T(0.430)	T/T(0.102)	0.532	G(0.683)	T(0.317)	0.921	0.780∼1.087	0.332
	Con	0.486	0.429	0.085		0.700	0.300			
rs5576	MDD	T/T(0.999)	C/T(0.001)		0.979	C(0.001)	T(0.999)	0.963	0.06∼15.419	0.979
	Con	0.998	0.002			0.001	0.999			

To determine the relationship between SNPs and the incidence of MDD, both patients and healthy controls were divided into two groups according to gender. The results from 376 female patients with MDD and 360 female healthy controls indicated a significantly decreased frequency of genotype A/A in rs16139 (Supplemental table S4), but increased genotype A/G (*χ* ^2^ = 11.423, *p* = 0.001). Meanwhile, the G-allele in rs16139 occurred at a low frequency and the A-allele occurred at a high frequency (OR = 0.071, 95% CI = 0.009–0.538, *χ* ^2^ = 11.300, *p* = 0.0008). Results from 324 male patients with MDD and 313 healthy controls suggested a significantly decreased frequency of genotype A/A in rs16139 (Supplemental table S5), but increased genotype A/G (*χ* ^2^ = 11.366, *p* = 0.008). Simultaneously, the G-allele in rs16139 occurred at a high frequency and the A-allele occurred at a low frequency (*χ* ^2^ = 11.256, *p* = 0.008).

### Linkage disequilibrium and haplotype analysis

Linkage disequilibrium analysis was performed on the 10 SNPs of NPY using the Haploview software. Eight closely linked SNPs in NPY were detected between rsl6147 and rsl6478, rs16135 and rs5574, rs5574 and rsl6129, rsl6135 and rsl6129, rsl6129 and rsl6147, rs16147 and rs3025118, rsl6147 and rs16135, as well as rsl6147 and rs5574 (1≥D’>0.8). According to the D value from the linkage disequilibrium analysis, 10 NPY SNPs were divided into two blocks and their correlation with MDD was analyzed. Correlation analysis showed no relationship between the incidence of MDD and the haplotype of NPY.

### Results of verification test with independent samples

We used independent samples to verify the relationship between SNPs of NPY and the pathogenesis of MDD. The results also showed that the gene polymorphism loci rs16139 was highly correlated with the onset of MDD (Table 2).

**Table 2:** The correlation analysis between the rs16139 locus of NPY and the susceptibility of MDD in independent samples.

Genotype or allele	Cases (%)	Controls (%)	OR	95%CI	*P*
AA	397(95.2)	312(99.4)	0.127	0.030−0.549	0.0011
AG	20(4.8)	2(0.6)			
A	814(97.6)	626(99.7)	0.130	0.030−0.558	0.0012
G	20(2.4)	2(0. 3)			

## Discussion

MDD is a common psychiatric disease with high levels of morbidity and mortality [8]. Despite intensive research efforts on the theoretical aspects of controlling MDD over the past several decades, the neurobiological basis and pathophysiology of depressive disorders remain unknown [9].

NPY is a highly evolutionarily conserved neuropeptide that is widely distributed in the central nervous system and participates in many neural regulations of physiological function [10]. Numerous studies have shown that NPY and depression are closely related with the human responses to stress [11], [12], [13]. Acute and chronic stresses are important factors influencing depression. In depression model rats induced by chronic unpredictable stress, many brain regions show downregulated expression of NPY [14], and the transgenic overexpression of NPY exerts a significant antidepressant-like effect [15]. Meanwhile, NPY knockout rats show significant depression and anxiety-like behavior [16]. Domschke K et al. found that NPY gene is involved in the human emotional process, i.e., allele variations in the polymorphism locus rs16147 affect the efficacy of antidepressant drugs [17]. Witt SH et al. found interactions between early life events and the polymorphism locus rs16147 in the promoter region of NPY gene. These interactions affect the responses of a patient’s hypothalamic-pituitary axis, ultimately leading to disease occurrence [18]. The polymorphism locus rs16147 of NPY also reportedly affects the mRNA expression and protein synthesis of NPY [19], [20].

A few reports have compared NPY in plasma or cerebrospinal fluid of patients with major depression or animal models. Most studies have found that the central NPY is insufficient in patients with depression, and that their NPY levels in plasma or cerebrospinal fluid are decreased [21], [22]. However, Hou et al. [23] compared the levels of NPY in cerebrospinal fluid in 40 cases of patients with major depression and healthy controls, and found no significant differences between them. Studies on the NPY levels in patients with major depression are not completely consistent, which can reflect the complexity of the etiology of depression. These studies have suggested that NPY is closely related to depression and its pathophysiology.

The results of this study showed that the polymorphism locus rs16139 of NPY gene was highly correlated with depression. This result was consistent with the results of Sjöholm LK et al [24]. Bosker FJ et al [25] in the GAIN MDD study also found the involvement of C5orf20 (rs12520799) and NPY (rs16139) in MDD constituted a direct replication at SNP level. The frequency of minimum T-allele in rs16147 obtained in this study (control vs. MDD: 30.5% vs. 31.8%) differed from those obtained by Heilig et al. [11] (control vs. MDD: 60.9 % vs. 47.9%). Heilig et al [11] found that the polymorphism locus Tll28C of NPY was significantly correlated with the disease, whereas T-399C of NPY had no significant association. The -399C allele site in rs16147 was closely related to the curative effect and prognosis of depression. The 4 week clinical recovery rate was low in patients with a low frequency of -399C allele, whereas the rs16139 locus was not related to the curative effect and prognosis of depression [17]. Our study found that the frequency of allele Pro7 distributed in patients with major depression was high, whereas that in healthy controls was relatively low (control vs. MDD: 0.1% vs. 1.9%, OR = 0.039, *p* = 2.2×10^−6^), which differed from other previous findings. Allele Pro7 is a protective factor of depression [21], and the difference may be related to different population. The subject of our study was Chinese Han population. The distribution of allele Pro7 in the polymorphism locus rs16139 of NPY gene varies among different human populations. The frequency of allele Pro7 is significantly higher in Nordic population than in other populations [26].

In conclusion, our results provided important information for understanding the relationship between SNPs of NPY and the susceptibility to depression. A significant correlation was observed between the SNP sites rs16139 in NPY and the morbidity of depression. Moreover, patients with MDD had a low frequency of A-allele and a high G-allele in rs16139. However, this frequency varied between male and female patients. These results were verified with independent samples. Therefore, the gene polymorphism loci rs16139 of NPY was closely related to the onset of depression, although its role in the pathogenesis of MDD requires further study.

## Supporting Information

Table S1 **Primers Used in the Polymerase Chain Reaction–Ligase Detection Reaction Protocol.**
(DOC)

Table S2 **Probes Used in the Polymerase Chain Reaction–Ligase Detection Reaction Protocol.**
(DOC)

Table S3 **NPY SNPs detection and HWE test from Chinese Han population.**
(DOC)

Table S4 **The correlation analysis between the Genotype and Alleles of NPY SNPs and the susceptibility of MDD in female.**
(DOC)

Table S5 **The correlation analysis between the Genotype and Alleles of NPY SNPs and the susceptibility of MDD in male.**
(DOC)
