# Correction: A Neuropeptide Y Variant (rs16139) Associated with Major Depressive Disorder in Replicate Samples from Chinese Han Population

**DOI:** 10.1371/annotation/f3ebdce9-783e-4e59-a3e8-d28369434fa5

**Published:** 2013-09-10

**Authors:** Yongjun Wang, Yutao Yang, Li Hui, Changle Tie, Feng Li, Zhi-Qing David Xu, Chuanyue Wang

Multiple funding organizations and grants were incorrectly omitted from the Funding Statement. The Funding Statement should read: "This work was supported by the Beijing Natural Science Foundation (Key Project, project number 09G0013), the Beijing Excellent Talent Project (project number PHR20100510), Tianjin Health Bureau Technology Fund(project number 2012KR01,12KG111) and the Beijing Key Laboratory of Major Brain Disorders (project number 2011NZDB01). The funders had no role in study design, data collection and analysis, decision to publish, or preparation of the manuscript." 

