# Supplementary material for: A Neuropeptide Y Variant (rs16139) Associated with Major Depressive Disorder in Replicate Samples from Chinese Han Population
Source: PLoS One. 2013 Feb 27;8(2):e57042. doi: 10.1371/journal.pone.0057042 (PMC3584142; doi:10.1371/journal.pone.0057042)
Supplement: Table S1 — Primers Used in the Polymerase Chain Reaction–Ligase Detection Reaction Protocol. (DOC) [file pone.0057042.s001.doc]

**Table S1. Primers Used in the Polymerase Chain Reaction–Ligase Detection Reaction Protocol**

| primers | Sequence(5’-3’) | length |
| --- | --- | --- |
| Rs16147 | CAGCTTTGGGACCCTCTAGC | 216bp |
|  | CTCCTGCCAACAGGACTACC |  |
| Rs16478 | CCTCTGCTCCACCTGAAAAC | 217bp |
|  | CCTGAAGCTAGGCAGACCAG |  |
| Rs16139 | CTGGTGTGCAGGCACTGG | 248 bp |
|  | CCTGCAGATGCTAGGTAACAA |  |
| Rs16138 | ACGACCACCAAAGAAAACCA | 243 bp |
|  | AGGGAGACCTCTGCATTTTAGA |  |
| Rs3025118 | CAGCATTGTAGAGGGGAAGG | 239 bp |
|  | TTCTGTCCATCCATTGGTTTT |  |
| Rs16135 | ACTCACTGGAGCATGATGAAGA | 208 bp |
|  | TGCTGAGACTCAAGGGAAGC |  |
| Rs5574 | TGAGCTCTGCAACAATGTCC | 166 bp |
|  | TGCCTATTCCAAACTTGCTTT |  |
| Rs6951110 | TTTTCTGCCTGTTTCCCTTG | 197 bp |
|  | CCCACCTTCCCTTCTAACCT |  |
| Rs16129 | TGCACCTGATGGAGTCTGTC | 241 bp |
|  | TGGCCAGGGTTGTCTTTAAC |  |
| Rs5576 | CTGCATGCATTGGTAGGATG | 152 bp |
|  | CCTTACATGCTTTGCTTCTTATG |  |
