# Supplementary material for: A Neuropeptide Y Variant (rs16139) Associated with Major Depressive Disorder in Replicate Samples from Chinese Han Population
Source: PLoS One. 2013 Feb 27;8(2):e57042. doi: 10.1371/journal.pone.0057042 (PMC3584142; doi:10.1371/journal.pone.0057042)
Supplement: Table S2 — Probes Used in the Polymerase Chain Reaction–Ligase Detection Reaction Protocol. (DOC) [file pone.0057042.s002.doc]

**Table S2. Probes Used in the Polymerase Chain Reaction–Ligase Detection Reaction Protocol**

| probes | Sequence(5’-3’) | length |
| --- | --- | --- |
| Rs16147R-modify | P-CCCACTGGGTGCCGGAGTAGGAAGCTTTTTTTTTTTTTTTTTTTT-FAM |  |
| Rs16147R-C | TTTTTTTTTTTTTTTTTTTTTGTCTCCTGCCAACAGGACTACCAG | 90 bp |
| Rs16147R-T | TTTTTTTTTTTTTTTTTTTTTTTGTCTCCTGCCAACAGGACTACCAA | 92 bp |
| Rs16478-modify | P-TCCCTGAGACTTCGATTTTTTTTTTTTTTTTTTTT-FAM |  |
| Rs16478-C | TTTTTTTTTTTTTTTTTTTTTTTGCACTCTCGCCGCGCGCTTCTTGG | 94bp |
| Rs16478-T | TTTTTTTTTTTTTTTTTTTTTTTTTGCACTCTCGCCGCGCGCTTCTTGA | 96bp |
| Rs16139-modify | P-GGGGCTGTCCGGACTGACCCTCGCCTTTTTTTTTTTTTTTTTTTTTTTTTTTT-FAM |  |
| Rs16139-A | TTTTTTTTTTTTTTTTTTTTTTTTTTTTTGCAGATGCTAGGTAACAAGCGACT | 106bp |
| Rs16139-G | TTTTTTTTTTTTTTTTTTTTTTTTTTTTTTTGCAGATGCTAGGTAACAAGCGACC | 108bp |
| Rs16138-modify | P-CTGCAGTTTACTCCTTGGTTTGGTTTTTTTTTTTTTTTTTTTTTTTT-FAM |  |
| Rs16138-C | TTTTTTTTTTTTTTTTTTTTTTTAAGTCTGACAATGTCTGTGGCAACG | 98bp |
| Rs16138-G | TTTTTTTTTTTTTTTTTTTTTTTTTAAGTCTGACAATGTCTGTGGCAACC | 100bp |
| Rs3025118R-modify | P-TCTTCTTCCTTCTTTAATTAATTCTTTTTTTTTTTTTTTTT-FAM |  |
| Rs3025118R-G | TTTTTTTTTTTTTTTTTTGCATCCAGTTCAGTACTGTTCAC | 82bp |
| Rs3025118R-T | TTTTTTTTTTTTTTTTTTTTGCATCCAGTTCAGTACTGTTCAA | 84bp |
| Rs16135- modify | P-AAATCTCACACATGATTTTTTTTTTTTTTTTTTTT-FAM |  |
| Rs16135-C | TTTTTTTTTTTTTTTTTTTTTCGCAACTCCAGAAA | 90bp |
| Rs16135-T | TTTTTTTTTTTTTTTTTTTTTTTCGCAACTCCAGAAAA | 92bp |
| Rs5574- modify | P-GATCGTTTTCCATATCTGGAAAAAATTTTTTTTTTTTTTTTTTTTTTTTTT-FAM |  |
| Rs5574-C | TTTTTTTTTTTTTTTTTTTTTTTTTTGTCTGAAATCAGTGTCTCTGGGCTG | 102bp |
| Rs5574-T | TTTTTTTTTTTTTTTTTTTTTTTTTTTTGTCTGAAATCAGTGTCTCTGGGCTA | 104bp |
| Rs6951110R- modify | P-TAAAATGGCCAGCCAGCATGAAGGGTTTTTTTTTTTTTTTTTTTTTTTT-FAM |  |
| Rs6951110R-C | TTTTTTTTTTTTTTTTTTTTTTTTCTTCTAACCTCCCTCAGTTCTCTTG | 98bp |
| Rs6951110R-G | TTTTTTTTTTTTTTTTTTTTTTTTTTCTTCTAACCTCCCTCAGTTCTCTTC | 100bp |
| Rs16129 -modify | P-CCTGATTGACAGACTTTTTTTTTTTTTTTTTTTTTTT-FAM |  |
| Rs16129 -G | TTTTTTTTTTTTTTTTTTTTTTGTGCATGAAGTAAAATTAATTGCAC | 94bp |
| Rs16129-T | TTTTTTTTTTTTTTTTTTTTTTTTGTGCATGAAGTAAAATTAATTGCAA | 96bp |
| Rs5576R- modify | P-TATTTCATCGTGTAAAACGAGAATCTTTTTTTTTTTTTTTTTT-FAM |  |
| Rs5576R-C | TTTTTTTTTTTTTTTTTTCTGGCCTTTTCCTATTTTCAGCCCG | 86bp |
| Rs5576R-T | TTTTTTTTTTTTTTTTTTTTCTGGCCTTTTCCTATTTTCAGCCCG | 88bp |
