# Supplementary material for: A Neuropeptide Y Variant (rs16139) Associated with Major Depressive Disorder in Replicate Samples from Chinese Han Population
Source: PLoS One. 2013 Feb 27;8(2):e57042. doi: 10.1371/journal.pone.0057042 (PMC3584142; doi:10.1371/journal.pone.0057042)
Supplement: Table S3 — NPY SNPs detection and HWE test from Chinese Han population. (DOC) [file pone.0057042.s003.doc]

**Table S3. NPY SNPs detection and HWE test from Chinese Han population**

| **SNP** | **Allele** | **Function** | **MAF** | **Call Rate** | **OHET）** | **E（HET）** | **p** |
| --- | --- | --- | --- | --- | --- | --- | --- |
| **rs16147** | **C/T** | **Promoter** | **0.311** | **98.3%** | **0.431** | **0.429** | **0.907** |
| **rs16478** | **C/T** | **Intron** | **0.253** | **97.6%** | **0.388** | **0.378** | **0.015** |
| **rs16139** | **A/G** | **extron** | **0.007** | **99.0%** | **0.015** | **0.015** | **1.000** |
| **rs16138** | **G/C** | **Intron** | **0.241** | **97.6%** | **0.364** | **0.366** | **0.959** |
| **rs3025118** | **G/T** | **Intron** | **0.024** | **98.6%** | **0.047** | **0.046** | **0.943** |
| **rs16135** | **A/G** | **Intron** | **0.295** | **98.3%** | **0.411** | **0.416** | **0.709** |
| **rs5574** | **C/T** | **Extron** | **0.382** | **97.3%** | **0.477** | **0.472** | **0.766** |
| **rs6951110** | **C/G** | **Intron** | **0.0** | **97.3%** | **0.0** | **0.0** | **1.000** |
| **rs16129** | **A/C** | **Intron** | **0.309** | **97.1%** | **0.429** | **0.427** | **0.879** |
| **rs5576** | **A/G** | **3‘UTR** | **0.001** | **98.9%** | **0.001** | **0.001** | **1.000** |

**Note: HWE: Hardy-Weinberg equilibrium；MAF：minor allele frequency；O（HET）：observed heterozygosity；E(HET): predicted heterozygosity；**
