# Supplementary material for: A Neuropeptide Y Variant (rs16139) Associated with Major Depressive Disorder in Replicate Samples from Chinese Han Population
Source: PLoS One. 2013 Feb 27;8(2):e57042. doi: 10.1371/journal.pone.0057042 (PMC3584142; doi:10.1371/journal.pone.0057042)
Supplement: Table S4 — The correlation analysis between the Genotype and Alleles of NPY SNPs and the susceptibility of MDD in female. (DOC) [file pone.0057042.s004.doc]

**Table S4. The correlation analysis between the Genotype and Alleles of NPY SNPs and the susceptibility of MDD in female**

| **SNPs ID** | **group** | **Genotype** | | | **P** | **Allele** | | **OR** | **95%CI** | **P** |
| --- | --- | --- | --- | --- | --- | --- | --- | --- | --- | --- |
| **rs16147** | **MDD** | **C/C（0.443）** | **C/T（0.451）** | **T/T（0.106）** | **0.369** | **C（0.702）** | **T（0.298）** | **0.861** | **0.688-1.007** | **0.189** |
|  | **Con** | **0.496** | **0.410** | **0.094** |  | **0.669** | **0.331** |  |  |  |
| **rs16478** | **MDD** | **C/C（0.537）** | **C/T（0.402）** | **T/T（0.061）** | **0.787** | **C（0.751）** | **T（0.249）** | **0.936** | **0.737-1.190** | **0.591** |
|  | **Con** | **0.562** | **0.377** | **0.061** |  | **0.739** | **0.261** |  |  |  |
| **rs16139** | **MDD** | **A/A（0.960）** | **A/G（0.040）** |  | **0.001** | **A（0.999）** | **G（0.001）** | **0.071** | **0.009-0.538** | **0.0008** |
|  | **Con** | **0.997** | **0.003** |  |  | **0.988** | **0.012** |  |  |  |
| **rs16138** | **MDD** | **C/C（0.063）** | **C/G（0.372）** | **G/G（0.565）** | **0.838** | **C（0.239）** | **G（0.761）** | **0.946** | **0.743-1.206** | **0.656** |
|  | **Con** | **0.064** | **0.351** | **0.586** |  | **0.251** | **0.749** |  |  |  |
| **rs3025118** | **MDD** | **G/G（0.931）** | **G/T（0.069）** |  | **0.238** | **G（0.973）** | **T（0.027）** | **0.692** | **0.370-1.293** | **0.246** |
|  | **Con** | **0.952** | **0.048** |  |  | **0.965** | **0.035** |  |  |  |
| **rs16135** | **MDD** | **C/C（0.499）** | **C/T（0.410）** | **T/T（0.091）** | **0.764** | **T（0.314）** | **C（0.686）** | **1.089** | **0.868-1.365** | **0.462** |
|  | **Con** | **0.490** | **0.416** | **0.094** |  | **0.314** | **0.686** |  |  |  |
| **rs5574** | **MDD** | **C/C（0.393）** | **C/T（0.487）** | **T/T（0.120）** | **0.748** | **C（0.618）** | **T（0.382）** | **1.080** | **0.870-1.1342** | **0.484** |
|  | **Con** | **0.374** | **0.488** | **0.137** |  | **0.636** | **0.364** |  |  |  |
| **rs6951110** | **MDD** | **C/C（1.000）** |  |  |  | **C（1.000）** |  |  |  |  |
|  | **Con** | **1.000** |  |  |  | **1.000** |  |  |  |  |
| **rs16129** | **MDD** | **G/G（0.433）** | **G/T（0.464）** | **T/T（0.103）** | **0.175** | **G（0.709）** | **T（0.291）** | **0.815** | **0.650-1.023** | **0.077** |
|  | **Con** | **0.503** | **0.412** | **0.085** |  | **0.665** | **0.335** |  |  |  |
| **rs5576** | **MDD** | **T/T（0.997）** | **C/T（0.003）** |  | **0.983** | **C（0.001）** | **T（0.999）** | **1.031** | **0.064-16.520** | **0.983** |
|  | **Con** | **0.997** | **0.003** |  |  | **0.001** | **0.999** |  |  |  |
