# Supplementary material for: A Neuropeptide Y Variant (rs16139) Associated with Major Depressive Disorder in Replicate Samples from Chinese Han Population
Source: PLoS One. 2013 Feb 27;8(2):e57042. doi: 10.1371/journal.pone.0057042 (PMC3584142; doi:10.1371/journal.pone.0057042)
Supplement: Table S5 — The correlation analysis between the Genotype and Alleles of NPY SNPs and the susceptibility of MDD in male. (DOC) [file pone.0057042.s005.doc]

**Table S5. The correlation analysis between the Genotype and Alleles of NPY SNPs and the susceptibility of MDD in male**

| **SNPs ID** | **Group** | **Genotype** | | | **P** | **Allele** | | **OR** | **95%CI** | **P** |
| --- | --- | --- | --- | --- | --- | --- | --- | --- | --- | --- |
| **rs16147** | **MDD** | **C/C（0.494）** | **C/T（0.408）** | **T/T（0.098）** | **0.494** | **C（0.698）** | **T（0.302）** | **1.048** | **0.823-1.333** | **0.706** |
|  | **Con** | **0.461** | **0.454** | **0.085** |  | **0.688** | **0.312** |  |  |  |
| **rs16478** | **MDD** | **C/C（0.575）** | **C/T（0.365）** | **T/T（0.060）** | **0.484** | **C（0.757）** | **T（0.243）** | **1.090** | **0.843-1.411** | **0.510** |
|  | **Con** | **0.535** | **0.412** | **0.053** |  | **0.741** | **0.259** |  |  |  |
| **rs16139** | **MDD** | **A/A（0.963）** | **A/G（0.037）** |  | **0.008** | **A（0.981）** | **G（0.019）** |  |  | **0.008** |
|  | **Con** | **1.000** | **0.000** |  |  | **1.000** | **0.000** |  |  |  |
| **rs16138** | **MDD** | **C/C（0.057）** | **C/G（0.360）** | **G/G（0.583）** | **0.827** | **C（0.237）** | **G（0.763）** | **1.023** | **0.785-1.332** | **0.868** |
|  | **Con** | **0.047** | **0.372** | **0.581** |  | **0.233** | **0.767** |  |  |  |
| **rs3025118** | **MDD** | **G/G（0.962）** | **G/T（0.038）** |  | **0.570** | **G（0.981）** | **T（0.019）** | **0.722** | **0.435-1.198** | **0.206** |
|  | **Con** | **0.970** | **0.030** |  |  | **0.985** | **0.015** |  |  |  |
| **rs16135** | **MDD** | **C/C（0.517）** | **C/T（0.404）** | **T/T（0.079）** | **0.937** | **T（0.281）** | **C（0.719）** | **1.038** | **0.810-1.329** | **0.768** |
|  | **Con** | **0.4510** | **0.403** | **0.087** |  | **0.302** | **0.698** |  |  |  |
| **rs5574** | **MDD** | **C/C（0.372）** | **C/T（0.458）** | **T/T（0.170）** | **0.799** | **C（0.601）** | **T（0.399）** | **0.943** | **0.750-1.186** | **0.615** |
|  | **Con** | **0.380** | **0.470** | **0.150** |  | **0.615** | **0.385** |  |  |  |
| **rs6951110** | **MDD** | **C/C（1.000）** |  |  |  | **C（1.000）** |  |  |  |  |
|  | **Con** | **1.000** |  |  |  | **1.000** |  |  |  |  |
| **rs16129** | **MDD** | **G/G（0.508）** | **G/T（0.390）** | **T/T（0.102）** | **0.327** | **G（0.703）** | **T（0.297）** | **1.062** | **0.832-1.356** | **0.630** |
|  | **Con** | **0.466** | **0.449** | **0.085** |  | **0.690** | **0.310** |  |  |  |
| **rs5576** | **MDD** | **T/T（1.000）** | **C/T（0.000）** |  |  | **C（0.000）** | **T（1.000）** |  |  |  |
|  | **Con** | **1.000** | **0.000** |  | **0.494** | **0.000** | **1.000** |  |  |  |
